# Distal Tibia Fractures: is the Tibia First Technique a Rational Approach?

**DOI:** 10.5704/MOJ.2303.020

**Published:** 2023-03

**Authors:** G Touloupakis, M Messori, A Gilli, E Theodorakis, S Ghirardelli, G Antonini

**Affiliations:** 1 Department of Orthopedics and Traumatology, San Carlo Borromeo Hospital, Milan, Italy; 2 Department of Orthopedics and Traumatology, Hospital Brixen Bressanone, Bressanone, Italy

**Keywords:** pilon fracture, tibia-first concept, osteosynthesis, trauma, minimally invasive plate osteosynthesis

## Abstract

**Introduction:**

In this retrospective case-series study we discuss the clinical and radiographic outcomes obtained following the “tibia-first concept” in the treatment of distal tibia fractures, both in patients with fibular comminution and in cases with a simple fibula fracture.

**Materials and methods:**

We analysed a consecutive series of 64 patients who presented at our emergency department with a distal articular tibial and fibular fracture from January 2015 to September 2020. A total of 22 patients met the inclusion and exclusion criteria and were included in the study. Clinical and radiographic examination were performed at each follow-up. To quantify pain and functional disability, the Foot and Ankle Outcome Score (FAOS) and the American Orthopaedic Foot and Ankle Society’s ankle-hindfoot scale (AOFAS) were applied.

**Results:**

The overall mean age was 52.8 years, and the mean follow-up was 13.18 months. Multiple scales data from the FAOS were as follows: pain score 80.70; symptoms score 81.69; activities of daily living score 87.22; quality of life 76.05. The mean AOFAS ankle-hindfoot score was 74.36.

**Conclusions:**

Even though the principles of Rüedi and Allgöwer are still valid, in specific circumstances, the tibia-first concept could be considered as a valid option for the treatment of these demanding fractures. If a good reduction is obtained intra-operatively by ligamentotaxis, we recommend fixing the tibia first, avoiding surgical stress on tissues derived from a previous fibular fixation.

## Introduction

Distal tibia fractures (DTF) mostly result from high-energy trauma and are often associated with a fibular fracture and severe soft tissue damage. Surgical treatment is challenging, and the surgeon deals with complications such as infections, delayed wound healing, implant failure, and post-traumatic arthritis on a regular basis. An accurate reduction of the articular surface is mandatory to gain a satisfactory clinical outcome, but the best timing for a surgical procedure is often dictated by the soft tissue conditions.

The trend in approaching these injuries is to perform a two-stage procedure. First a damage control surgery (DCS) with external fixation to address or prevent any soft tissue damage. Then, after an adequate soft tissue recovery, a definitive treatment can be performed by open reduction and internal fixation (ORIF).

According to Rüedi and Allgöwer, ORIF of distal tibia and fibular fractures with articular involvement should be performed by starting with the fibular fixation to gain adequate length and stability. Subsequently, anatomic reconstruction of the articular surface and osteosynthesis of the tibial pilon fracture can be performed^1,2.^ Recently, in case of comminuted fractures of the fibula, some authors suggest performing tibial ORIF as first step, since fibular comminution implies difficulties in achieving anatomic length and rotation. In such cases a malreduction of the fibula compromises the quality of the pilon fractures’ reduction^3^.

In this retrospective case-series we discuss functional and radiographic outcomes in patients treated following what we define the “tibia-first concept”. According to the tibia first concept, fractures of the distal one third of tibia and fibula were treated by fixing the tibia fracture first, regardless of fibula comminution, i.e. both in cases with a multifragmentary fibula and in cases with a simple fibular fracture.

## Materials and Methods

Ethical Considerations: the patients, after a consult with the surgical team, signed an informed consent to undergo a surgical procedure of internal fixation and to allow divulgation of anonymised images, radiographs and data for scientific purposes. From January 2015 to September 2020, 64 patients presented at our emergency department with a diagnosis of distal tibial and fibular fracture. After a preliminary review 22 met the inclusion criteria and were analysed.

Inclusion criteria were: (1) patients with distal fibular and distal tibial fracture; (2) patients who underwent standard radiograph and CT scan before surgery and (3) patients with a minimum of 12 months follow-up. The exclusion criteria were: (1) patients treated with intramedullary nailing or definitive external fixation; (2) paediatric patients (<18 years old); (3) patients with concomitant other fractures of ankle or foot and (4) patients with a history of previous ankle or foot surgery.

Demographic and clinical data were collected. Fractures were classified according to AO/OTA classification. Data on surgical treatments, approaches and type of synthesis were recorded. All patients were followed up as outpatients with radiographs according to our protocol (anteroposterior and lateral projection of leg and the ankle) at 5 weeks, 3 months, 6 months, 12 months, and since then every year. A proof clinical and radiographic examination was performed at each follow-up. Post-operative complications such as deep infections, neurovascular injuries, non-unions, malunions, malalignments and articular incongruence, were examined in the immediate post-operative period and at last follow-up. To quantify pain and functional disability, the Foot and Ankle Outcome Score (FAOS) and the American Orthopaedic Foot and Ankle Society’s ankle-hindfoot scale (AOFAS) were applied at the last follow-up.

One shot antibiotic prophylaxis with cefazolin or clindamycin was administered intravenously to every patient. Under general or spinal anaesthesia, patients were positioned supine on a radiolucent table. No tourniquet was used during the entire surgical procedure. In case of a severe swelling, blisters or an exposed tibial fracture, a two-stage treatment was performed. In those cases, an external fixator spanning the ankle joint was applied. After edema resolution and a positive wrinkle test, definitive fixation was performed 7 to 15 days after trauma.

In our pre-operative planning two scenarios were considered. On the one hand, if good alignment was achieved by ligamentotaxis (i.e. with the provisional external fixator, the distractor or transcalcaneal pin), patients were treated according to “tibia first” approach, which means that DTF were fixed before distal fibular fractures. On the other hand, when ligamentotaxis wasn’t sufficient to achieve good reduction and alignment our surgical strategy was based on the classical criteria of starting with the less comminuted fracture (either tibia or fibula). In this study only patients treated with tibia first approach were included.

Depending on the severity of the soft tissue swelling, open reduction and internal fixation (ORIF) or minimal invasive plate osteosynthesis (MIPO) techniques were performed. Anterolateral approach of the distal tibia was considered as first choice, while anteromedial or medial approach were considered in case of contraindications of the latter. The reductions were accurately confirmed by radiographs fluoroscopy, and fixations were obtained by anterolateral or medial locking compression plates (LCP). In cases of severe displacement authors opted for a double plating with both anterolateral and medial LCP plates.

After tibial fixation, fibular fractures were treated by lateral approach. Depending on the grade of the soft tissue swelling, open reduction and internal fixation (ORIF) or minimal invasive plate osteosynthesis (MIPO) techniques were performed. MIPO technique was considered as first choice, while ORIF technique was chosen in case of non-satisfying indirect reduction. Surgical wounds were closed in a standard fashion. A negative pressure wound dressing was used in cases of severe soft tissue swelling based on surgeon preferences.

Post-operative functional exercises were allowed the day after surgery without any restriction of limb movement. Weight-bearing exercise was prohibited for the first four weeks. At four weeks post-surgery, gradual partial weight-bearing exercises of the lower limb were carried out under the guidance of the physician. Full weight-bearing was allowed 8-10 weeks after the surgery.

## Results

**Fig. 1:**
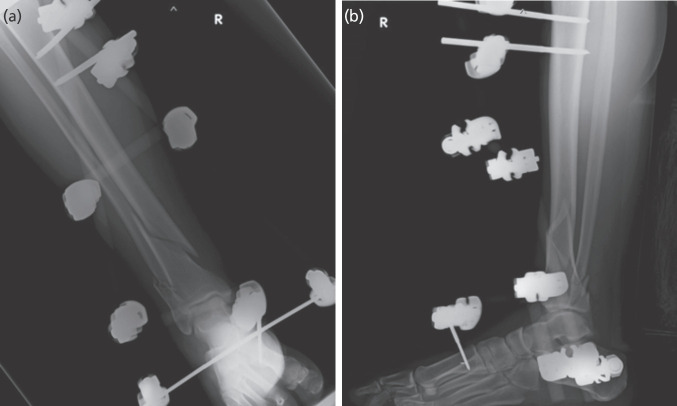
Radiographs of the ankle joint after stabilisation with external fixator. (a) Antero-posterior view. (b) Lateral view.

**Fig. 2:**
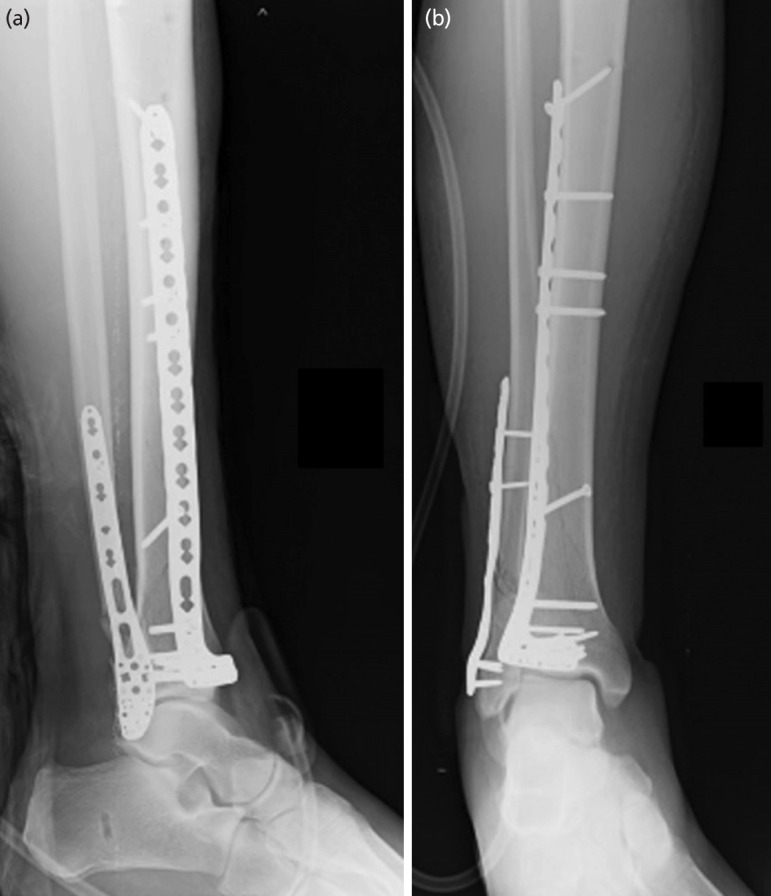
Post-operative radiographs. (a) Lateral view. (b) Antero-posterior view.

**Fig. 3:**
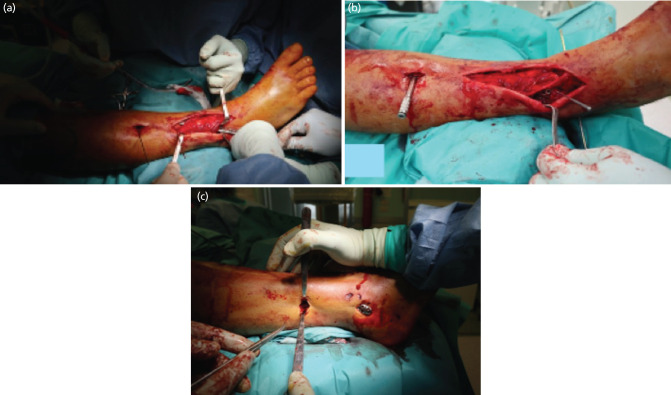
(a) Intra-operative condition: anterolateral approach of the distal tibia. (b) Identification of the superficial peroneal nerve. (c) MIPO technique for fibula fixation.

**Fig. 4:**
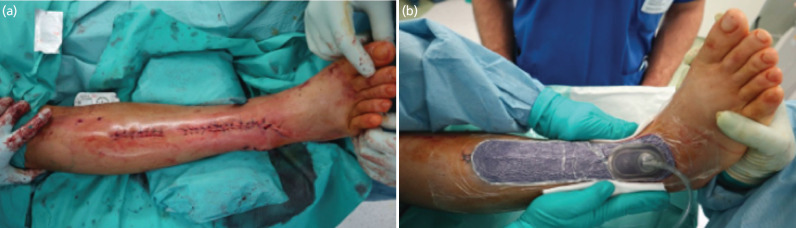
(a) Skin closure of the anterolateral approach of the distal tibia. (b) Application of the PREVENA™ [KCI USA, Inc., San Antonio, TX] incision management system to protect the incision from external contamination.

Twenty-two patients were enrolled, sixteen male and six female. The overall mean age was 52.8 years ± 19.2 (25 to 88) and the mean follow-up was 13.18 months ± 3.19 (12 to 24). Nine patients sustained a low energy trauma, while thirteen patients were involved in motor vehicle accidents (Fig. 1-4). The AO/OTA classification of the fractures is summarised in Table I. Surgical data, surgical approaches and surgical technique are described in Table II. Negative pressure wound dressing was used in two cases.

**Table I:** Demographic and pre-operative characteristics of the study population.

Parameter	Value	Percentage
Age (years)*	52.3 (25 to 88)	/
Sex		
Male	16	72%
Female	6	28%
Mechanism of injury		
Low energy	9	41%
High energy	13	59%
AO/OTA Classification		
43 A1 + 4F3 A	6	27%
43 A2 + 4F3 B	3	14%
43 A3 + 4F3 B	3	14%
43 A3 + 4F3 A	2	9%
43 C3 + 4F3 B	2	9%
43 A1 + 4F3 B	1	4.5%
43 A2 + 4F3 A	1	4.5%
43 B1 + 4F3 B	1	4.5%
43 C1 + 4F3 A	1	4.5%
43 C1 + 4F3 B	1	4.5%
43 C2 + 4F3 B	1	4.5%
Follow-up (mo)*	13.18 (12 to 24)	/

*The values are presented as the means and the range

**Table II:** Intra-operative data of the study population.

Surgical treatment	N	%
Single stage	18	82%
Two-stage	4	18%
Tibial approach		
Single approach	19	86%
Anteromedial	17	78%
Anterolateral	2	8%
Double approach		
Medial / Anteromedial (MIPO) + Anterolateral	3	14%
Tibial surgical technique		
ORIF	7	32%
MIPO	15	68%
Fibular surgical technique		
ORIF	18	82%
MIPO	4	18%

Abbreviations - ORIF: Open Reduction Internal Fixation, MIPO: Minimal Invasive Plate Osteosynthesis

FAOS scores were as follows: pain score = 80.70±14.01, symptoms score = 81.69±11.53, activities of daily living score = 87.22±10.02, quality of life = 76.05±18.69; sport score = 65.45±23.95. The mean AOFAS ankle-hindfoot score was 74.36±18.59 (Table III). Complications were recorded in three patients: one of them had superficial infection that responded to antibiotic therapy with no need of further surgical treatment and another two patients showed malalignment at the last follow-up: 14° varus deformity in one patient and recurvatum deformity in the other one. None of them was considered amenable to surgical correction.

**Table III:** Functional outcomes at last follow-up of the study population.

Age(y)/Sex			FAOS*			Quality of Life
	Pain	Symptoms	Activities of Daily Living	Sport	AOFAS*	
38/M	85.41	89.29	98.54	90	100	100
52/M	71.17	67.86	76.76	75	81	87
78/M	66.67	64.12	73.36	35	61	62
30/M	91.49	82.14	97.06	95	87	86
73/F	53.72	62.67	73.56	30	56	45
88/M	62.12	64.29	75.47	35	49	52
73/F	67.78	71.23	75.71	40	42	54
25/M	94.44	92.86	94.12	85	87	92
83/M	63.22	67.86	73.56	25	42	45
80/F	61.89	72.42	73.56	30	49	42
41/M	97.11	89.29	95.59	50	87	78
55/F	88.89	90.34	92.65	75	87	86
42/M	83.45	85.56	98.53	75	74	81
29/M	96.21	100	100	95	100	100
29/M	97.87	92.86	93.67	95	100	98
37/M	91.23	85.71	91.18	90	94	87
47/M	85.47	89.45	88.67	80	87	83
56/M	83.45	85.71	89.71	70	81	71
48/F	85.32	94.71	92.65	75	87	82
47/M	93.21	88.24	98.53	70	74	79
66/F	63.67	71.92	77.67	50	67	54
47/M	91.57	88.74	88.24	75	81	72
Mean	80.70	81.69	87.22	65.45	76.05	74.36

Abbreviations - FAOS: Foot Ankle Outcome Score, AOFAS: American Orthopaedic Foot and Ankle Society scale

## Discussion

DTF associated with distal fibular fracture were classically treated by restoring limb length through the fibular reduction first and then fixing the tibial fracture^1,3^. However, we are convinced that “tibia first” approach is more useful and safer in several cases. In case of great fibular comminution, the anatomic reduction is difficult to achieve due to its fracture pattern. Subsequent fibular malreduction in terms of length, axis and rotation could compromise tibial fixation as a second step^4,5^.

In our experience in complex pilon fractures ligamentotaxis has a greater impact on the distal tibia than on the distal fibula. This is evident when using a spanning external fixator in the setting of damage control orthopaedics, where traction and external manoeuvres usually restore tibial alignment while leaving a residual displacement of the fibula^6^. In addition, approaching DTF as a second step after the fibular fixation, may increase soft tissue swelling. A surgical incision on the distal tibial after surgical treatment of the peroneal fracture acts as a further damage to soft tissues which have been already stressed.

Based on our experience, the general principle of treating distal tibial-fibular fractures starting from the less comminuted bone should be revised. In those tibial fractures (either simple or comminuted) where the correct tibial length and alignment can be achieved intra-operatively by ligamentotaxis, the surgeon may perform tibial osteosynthesis first. Ligamentotaxis is obtained either with a provisional external fixator, a distractor or simply with a transcalcaneal pin. This strategy will avoid further stresses on soft tissues caused by a previous incision over the fibula.

As a further confirmation of our thesis, recent literature suggests avoiding treating fibula fracture in a complex DTF scenario^4,6-8^. These papers showed that there is no difference in final alignment when comparing fibular fractures with or without fixation in non-rotational pilon injuries. The authors underline the importance of distal tibia fixation as the key for a good functional result with a lower risk of complications such as non-union or infections. While taking into consideration the authors findings, we still assume fibular fixation helps to maintain axial alignment as confirmed by literature but at the same time we profoundly consider treatment of DTF before fibular fracture is safer and more efficient after all^9,10^. Regarding the fibular surgical technique, we consider fibular MIPO technique less demanding to restore length, axis and rotation after treating tibial fracture^11,12,13^.

According to the tibia first concept, anteromedial and/or anterolateral approaches are the surgical approaches of choice. They provide a good anatomic exposure of the distal tibial metaphyseal and articular fragments^14,15^. We also suggest an overzealous CT based pre-operative planning to choose the right approach which best suits the tibial fracture pattern^16,17^.

Our clinical outcomes were comparable to the literature. Jansen *et al* calculated a mean AOFAS score of 65 points in high energy DTF fractures, while Ketz *et al* found the AOFAS score as 85.2 on average in patients treated with posterior approach and 76.4 in patients operated with a standard anterior approach^18,19^. The FAOS score published by Zheng *et al*^20^ in 27 pilon fractures was as follow: pain score, 88.79±8.59; activities of daily living score, 91.89±7.50; quality of life score 90.26±10.52; sport score, 87.93±11.64; symptoms score, 85.32±8.65, whereas mean AOFAS score was 87.30±13.45. Compared to literature, our study exhibits satisfying results in AOFAS score, pain and symptoms scores of FAOS scale, showing that tibia-first concept could lead to good clinical and functional outcomes. Activities of daily living, quality of life, and sport scores were lower compared to literature, since functional outcomes related to other variables (i.e., age of patient, degree of joint involvement, etc.)

This study has several limitations. First, the limited number of cases. Second, the retrospective design. Third, two surgeons were involved in this study and, although both follow the tibia first concept, the choice of the surgical approach on the tibia and fibula still relies on personal judgement. Finally, for some patient long-term functional outcome is lacking.

## Conclusion

DTF are complex fracture that requires surgical experience and a careful evaluation of soft tissues. Even though the principles of Rüedi and Allgöwer are still valid, they need to be modified based on the fracture pattern. In some circumstances, previously described in this study, the tibia-first concept is a valid and safe approach that avoid excessive soft tissue insult and achieve excellent results. In conclusion, based on our experience tibia first technique is a rational approach regardless of the extent of fibula and tibial comminution. If a good reduction is obtained intra-operatively by ligamentotaxis we recommend fixing the tibia first, avoiding thus surgical stress on tissues derived from a previous fibular fixation.
